# Non-autoimmune subclinical hypothyroidism due to a mutation in TSH receptor: report on two brothers

**DOI:** 10.1186/1824-7288-39-5

**Published:** 2013-01-19

**Authors:** Manuela Cerbone, Patrizia Agretti, Giuseppina De Marco, Nicola Improda, Claudio Pignata, Francesca Santamaria, Massimo Tonacchera, Mariacarolina Salerno

**Affiliations:** 1 Department of Pediatrics, University of Naples “Federico II”, Naples, Italy; 2 Department of Endocrinology and Metabolism, University of Pisa, Pisa, Italy

**Keywords:** Subclinical hypothyroidism, Congenital hypothyroidism, TSH receptor mutation, Growth

## Abstract

Subclinical hypothyroidism (SH) is a condition characterized by a mild persistent thyroid failure. The main cause is represented by autoimmune thyroiditis, but mutations in genes encoding proteins involved in TSH pathway are thought to be responsible for SH, particularly in cases arising in familial settings. Patients with the syndrome of TSH unresponsiveness may have compensated or overt hypothyroidism with a wide spectrum of clinical and morphological alterations depending on the degree of impairment of TSH-receptor (TSH-R) function. We describe the case of two brothers with non autoimmune SH carrying the same heterozygous mutation in the extracellular domain of TSH-R and presenting with different clinical, biochemical and morphological features. The first one had only a slight persistent elevation of TSH, a normal thyroid ultrasound and did never require l- thyroxine (L-T4) replacement treatment. The second one had a neonatal persistent moderate TSH levels increase associated with a thyroid gland hypoplasia and was treated with L-T4 since the first months of life.

These two cases support the recent association of TSH-R mutations inheritance as an autosomal dominant pattern with variable expressivity and suggest that the decision to start replacement therapy in patients with persistent SH due to TSH resistance should be individualized.

## Background

Subclinical hypothyroidism (SH) is a biochemical condition characterized by serum levels of TSH above the statistically defined upper limit of reference range, with normal concentrations of thyroid hormones and without severe clinical features of hypothyroidism [1]. The prevalence has been reported to be between 4 and 20% of the adult population [2] and about 1.7% in US children [3]. However, in childhood it seems to be a benign remitting condition with a low risk of progression to overt hypothyroidism [1,4-8]. During neonatal period, a slight increase in TSH levels with normal thyroid hormone may be transient and sometimes be the cause of a false positive at neonatal screening for congenital hypothyroidism (CH) [9]. However, in other cases it may also be the expression of a mild persistent thyroid failure due to genetic abnormalities of thyroid structure or function. The most frequent cause of persistent SH in childhood is represented by autoimmune thyroiditis. However, iodine deficiency, obesity, non-thyroidal chronic diseases or inherited syndromes may also be responsible for mild increase of TSH levels. When no overt causes of slight TSH increase are detectable the condition is defined as idiopathic SH [10]. Mutations in genes encoding proteins involved in TSH pathway are thought to be responsible for some cases of idiopathic SH, particularly in cases arising in familial settings [11,12]. TSH exerts its activity by binding to the extracellular domain of TSH receptor (TSH-R). TSH-R is a member of the G protein-coupled receptor family that also includes calcitonin and PTH receptors [13]. It mediates the effects of TSH in thyroid development, growth, and thyroid hormone synthesis. Here we describe the case of two brothers with non autoimmune SH carrying the same TSH-R mutation and presenting with different clinical, biochemical and morphological features.

## Case presentation

Patient 1 was a young boy admitted to our Pediatric Endocrinology Unit at the age of 8-years for the finding of isolated hyperthyreotropinemia. He was the first child of healthy unrelated parents. Two blood samples performed three months apart showed a slight increase in TSH levels (TSH: 8 μU/ml, normal range: 0.5-4.5) with normal FT4 concentration (FT4 1.2 ng/dl, normal range: 0.7-1.6). The neonatal history was normal, he was not affected by any chronic disease, and was not taking any drugs potentially interfering with thyroid function. The reason for thyroid function screening was the familiarity for thyroid diseases. In fact, the younger brother was affected by CH and was treated with L-T4 since he was 4-months-old. The physical examination was normal, no signs or symptoms of hypothyroidism goiter included were detectable. Auxological parameters were normal, both weight and height being between 50th-75th percentile; height was appropriate to the genetic familial target. Autoimmune thyroiditis, morphological anomalies of thyroid gland, iodine deficiency and pseudohypoparathyroidism, were excluded by clinical, biochemical and ultrasound evaluation. He was not treated with L-T4 due to the slight elevation of TSH (ranging always between 4.5 and 10 μU/ml) and was closely monitored with clinical and laboratory examinations. At the age of 10 years, because of the persistence of SH and the familiarity for thyroid diseases, molecular analysis for TSH-R was performed. Direct sequencing of the extracellular and transmembrane-coding regions of the TSH-R showed an heterozygous missense mutation (CGG to CAG) in the exon 4 corresponding to an arginine to glutamine change at codon 109 (R109Q) in the extracellular domain of the receptor. The patient is now 18 years old. During the follow-up, despite a persistent slight elevation of TSH, he never required L-T4 replacement therapy. He always presented a normal growth and bone maturation. Puberty began and progressed regularly and the evaluation of intellectual outcome at the age of 16 years was normal with an intellectual quotient (IQ) of 108.

Patient 2, the younger brother of patient 1, was referred to our Unit at the age of 8 years for CH. The diagnosis of CH was performed on the basis of persistent moderate hyperthyreotropinemia (TSH 12–15 μU/ml) with low-normal values of FT4 (FT4 0.8 ng/dl; normal values 0.7-1.7), and a mild gland hypoplasia at thyroid ultrasound, incidentally discovered during a laboratory evaluation for growth retardation. The treatment with L-T4 was started at the age of 4 months with an initial dose of 6 μg/kg/day. Due to the finding of a TSH-R mutation in the older brother, we decided to re-evaluate the diagnosis of CH. The TSH-R molecular analysis showed the same heterozygous missense mutation of the brother, thus we decided to stop L-T4 therapy. However, TSH levels raised in a month over 10 μU/ml and persisted elevated in the following 3 measurements, ranging between 13 and 18 μU/ml. Moreover, he reported clinical symptoms of hypothyroidism such as fatigue, mood changes and impaired concentration in addition to significant weight gain. Therefore, L-T4 therapy was promptly re-started. During the follow-up he presented a normal growth and bone maturation. The puberty began and progressed regularly but the evaluation of IQ at the age of 16 years was slightly low (IQ 80).

## Discussion

Subclinical hypothyroidism is a relatively common condition characterized by a mild thyroid failure. It can occur at any age from neonatal period to adulthood in both transient or persistent form. Newborns with SH may have a transient impairment of thyroid function due to environmental or mother-derived causes, but they can also have genetic abnormalities of thyroid structure and function. During childhood the main cause of SH is generally represented by autoimmune SH, other known causes being mild developmental thyroid abnormalities, iodine deficiency, obesity, non-thyroidal chronic diseases or inherited syndromes and mutations in the TSH-R gene. Loss of function mutations in genes encoding proteins involved in the TSH pathway, TSH-R anomalies or alterations in the proteins involved in the signaling pathway downstream the receptor, have been widely demonstrated to be responsible of SH [11,14-18]. In a recent study, *Rapa et al.* evaluated the clinical characteristics and TSH-R gene variation in a series of children with slight to moderate elevation of TSH with normal thyroid hormone due to non autoimmune SH. Non-synonymous mutations of TSH-R gene or polymorphisms were found in 21.5% of cases [14]. The prevalence of a positive family history of thyroid diseases was two-fold higher in patients with mutations than in those with no mutations. An even higher prevalence of mutation in the TSH-R gene (29%) was detected by *Nicoletti et al.*[11]. Eleven mutations of TSH-R gene were identified in a cohort of 38 children and adolescents with non-autoimmune SH. In our first patient the main causes of persistent SH were ruled out at first examination. The diagnosis of partial resistance to TSH was suggested by the familiar history of thyroid diseases. Although both sibs were carrying the same mutation, they had completely different clinical phenotype. The first boy had a slight persistent increase of TSH (less than 10 μU/ml), normal thyroid ultrasound and did not show any signs or symptoms of mild hypothyroidism requiring L-T4 treatment. Conversely, the younger brother presented a moderate increase of TSH with serum levels persistently above 10 μU/ml with low normal serum FT4 concentrations, morphological thyroid anomalies with a mild thyroid hypoplasia. He needed to be treated in early infancy and relapsed when treatment was withdrawn. Indeed, even with a mild increase in TSH levels, he presented slight symptoms of hypothyroidism that prompted us to restore L-T4 treatment. Despite treatment, his IQ was slightly low thus raising the question on whether the delay in starting replacement treatment or a low initial dose of L-T4 may have influenced the suboptimal IQ outcome [19]. Pituitary TSH is critical for thyroid development and function and exerts its activity by binding to the extracellular domain of TSH-R, a G protein-coupled seven-transmembrane domain receptor located in the basolateral membrane of thyroid follicular cells. The principal biological effects of TSH on the thyrocite occur by receptor-mediated activation of Gsα and subsequent generation of intracellular cAMP [20,21]. The human TSH-R gene is located on chromosome 14q31 and encodes a protein of 764 amino acids. The gene is comprised of 10 exons, with the first nine encoding the extracellular domain and the large exon 10 encoding the transmembrane domain and the cytoplasmic tail [14]. Patients with the syndrome of TSH unresponsiveness may have compensated [17,22-24] or overt hypothyroidism [25-30] depending on the presence of a partial or complete TSH resistance. These conditions include a wide spectrum of biochemical, as well as clinical and morphological alterations depending on the degree of impairment of TSH-R function. The metabolic consequences can range from mild SH to severe CH. TSH is a prerequisite not only for normal thyroid hormone synthesis but also for proliferation. Accordingly, the inherited CH in the hyt/hyt mouse was shown to be caused by homozygosity for a loss of function mutation of the TSH-R (P556L) leading to thyroid hypoplasia and severe CH [29]. The mutation R109Q in the extracellular domain of TSH-xR, found in our two brothers, was first described by *Clifton-Bligh et al.* in a child compound heterozygote for another missense mutation in the fourth transmembrane segment of receptor. This child presented with markedly increased serum TSH concentrations and low normal thyroid hormone levels identified after a positive result on neonatal screening for CH, normal thyroid morphology and treated with L-T4 since 8 weeks of age [17]. Similarly to our second patient, after the L-T4 withdrawal at the age of 2 years, he showed a significant increase of TSH levels that required a resumption of therapy. Moreover, the R109Q mutation has also been documented in Italian children with non-autoimmune SH [11,14]. In particular, the two siblings reported by Nicoletti et al. [11], carrying the same heterozygous R109Q loss of function mutation found in our two patients, had a mild phenotype characterized by a slightly increase in serum TSH, normal thyroid gland at ultrasound and no clinical features of thyroid dysfunction. Similarly to our first patient they do not need treatment, thus highlighting the wide phenotypic variability in children with SH due to the same TSH-R mutation. In the earlier studies in which only probands with large TSH elevations were screened for mutations, the disease was linked to homozygous or compound heterozygous mutations and was described to follow a recessive pattern of inheritance [17,18,20-29]. More recently inheritance of TSH-R resistance has been associated with an autosomal dominant pattern without evidence for incomplete penetrance, but with variable expressivity. Several families have been described with dominant transmission of partial TSH resistance due to heterozygous inactivating mutations in TSH-R [30,31]. The recent finding that TSH-R, like other G protein-coupled receptors, can oligomerize in living cells led to the hypothesis that the formation of complexes between wild-type and mutant receptors may be responsible for partial TSH resistance in patients with heterozygous TSH-R mutations [31]. The clinical heterogeneity in our 2 patients carrying the same mutation, is not surprising as other monogenic diseases have been already shown to have a different phenotype despite the same causing mutation [32-36]. For most of these conditions, molecular basis of this heterogeneity are not well delineated. Disease modifying genes, variation in environmental exposures, as well as system dynamics may come into play in modulating clinical expression of the disease. If the treatment should be indicated or not in patients with only slight elevation of TSH due to TSH-R resistance is controversial. In fact, the elevation of circulating TSH would represent the compensatory mechanism allowing gland development and the maintenance of a normal thyroid hormone secretion in the presence of partial refractoriness to TSH action. Currently, there is no evidence that children with only slight increase in TSH levels (less than 10 μU/ml) and normal FT4 concentrations suffer any clinical or neurological abnormality that would be eventually restored to normal by L-T4 treatment [6,8]. Therefore the decision to treat with L-T4 SH still represents a clinical dilemma.

## Conclusions

Patients with persistent non autoimmune hyperthyreotropinemia should be investigated for TSH-R gene, in particular in cases arising in familial settings, even though the majority of patients with an heterozygous TSH-R loss of function mutation have only mild SH generally not requiring any treatment. However, it should be mentioned that some cases may have a more severe clinical presentation with the need of lifelong treatment. Currently is not possible to predict the clinical presentation and the evolution of these patients based on genetic features.

## Consent

Written informed consent was obtained from the parents of the patient for publication of this Case report and any accompanying images. A copy of the written consent is available for review by the Editor-in-Chief of this journal.

## Abbreviations

SH: Subclinical hypothyroidism; TSH-R: TSH-receptor; L-T4: l- thyroxine; CH: Congenital hypothyroidism; IQ: Intellectual quotient.

## Competing interests

The authors declare that they have no competing interests.

## Authors’ contributions

All authors have equally participated in drafting of the manuscript and/or critical revision of the manuscript for important intellectual content. All authors read and approved the final manuscript.
